# The unmet need for Emergency Obstetric Care in Tanga Region, Tanzania

**DOI:** 10.1186/1471-2393-7-16

**Published:** 2007-08-06

**Authors:** Helen Prytherch, Siriel Massawe, Rainer Kuelker, Claudia Hunger, Ferdinand Mtatifikolo, Albrecht Jahn

**Affiliations:** 1 Tanzanian German Programme to Support Health, Dar-es-Salaam, Tanzania; 2 Muhimbili National Hospital, Obstetrics & Gynaecology Department, Dar-es-Salaam, Tanzania; 3 Bombo Regional Hospital, Head of Obstetrics & Gynaecology, Tanga, Tanzania; 4 Department of Tropical Hygiene and Public Health, Ruprecht-Karls-University, Heidelberg, Germany

## Abstract

**Background:**

Improving maternal health by reducing maternal mortality constitutes the fifth Millennium Development Goal and represents a key public health challenge in the United Republic of Tanzania. In response to the need to evaluate and monitor safe motherhood interventions, this study aims at assessing the coverage of obstetric care according to the Unmet Obstetric Need (UON) concept by obtaining information on indications for, and outcomes of, major obstetric interventions. Furthermore, we explore whether this concept can be operationalised at district level.

**Methods:**

A two year study using the Unmet Obstetric Need concept was carried out in three districts in Tanga Region, Tanzania. Data was collected prospectively at all four hospitals in the region for every woman undergoing a major obstetric intervention, including indication and outcome. The concept was adapted to address differentials in access to emergency obstetric care between districts and between rural and urban areas. Based upon literature and expert consensus, a threshold of 2% of all deliveries was used to define the expected minimum requirement of major obstetric interventions performed for absolute maternal indications.

**Results:**

Protocols covering 1,260 complicated deliveries were analysed. The percentage of major obstetric interventions carried out in response to an absolute maternal indication was only 71%; most major obstetric interventions (97%) were caesarean sections. The most frequent indication was cephalo-pelvic-disproportion (51%). The proportion of major obstetric interventions for absolute maternal indications performed amongst women living in urban areas was 1.8% of all deliveries, while in rural areas it was only 0.7%. The high proportion (8.3%) of negative maternal outcomes in terms of morbidity and mortality, as well as the high perinatal mortality of 9.1% (still birth 6.9%, dying within 24 hours 1.7%, dying after 24 hours 0.5%) raise concern about the quality of care being provided.

**Conclusion:**

Based on the 2% threshold, Tanga Region – with an overall level of major obstetric interventions for absolute maternal indications of 1% and a caesarean section rate of 1.4% – has significant unmet obstetric need with a considerable rural-urban disparity. The UON concept was found to be a suitable tool for evaluating and monitoring the coverage of obstetric care at district level.

## Background

The fifth Millennium Development Goal set for 2015 aims to improve maternal health through a reduction of the maternal mortality ratio by three quarters and presents us with a key public health challenge [1]. In Tanzania, the maternal mortality ratio is high with estimates ranging from 529 to 1,500 maternal deaths per 100,000 live births [2-4]. In contrast, we find that coverage of antenatal care (ANC) is almost complete (97%) and that the health infrastructure is relatively well developed with 90% of the population living within 10 km of a health facility providing antenatal and delivery care. Furthermore, demographic and health survey figures show that 47% of deliveries are taking place in health facilities [4]. Despite these figures and conditions (high maternal mortality ratio, high ANC attendance rate, comparatively well-developed primary health care infrastructure, and a fair percentage of professionally monitored deliveries) a substantial improvement in maternal mortality reduction remains elusive. Therefore, the need arises to look deeper into the accessibility and quality of obstetric services in Tanzania. The complexity of gathering data about maternal mortality is widely acknowledged. The conventional approach of monitoring the level of maternal mortality using rates and ratios as indicators is inherently complex; figures suffer from under-reporting and misclassification whilst the methods required to gather the data are unwieldy and require substantial resources [5]. A systematic WHO review undertaken in 2006 underlined the persisting paucity of reliable data from sub-Saharan Africa and brings renewed attention to the ongoing need for improved data on the causes of maternal death at the country level [6].

A set of process indicators have been proposed by WHO, UNICEF and UNFPA to monitor interventions aimed at reducing maternal mortality. The six indicators include the crude proportion of caesarean sections among all births [7]. The Unmet Obstetric Need (UON) concept takes this idea further by including all major obstetric interventions and then stratifying them according to indications. We chose to use this service-orientated approach, developed by the UON network [8], as it can be applied at the lower levels of the health system and makes direct use of local data collected by health staff themselves.

The UON concept uses absolute maternal indications (AMI) with known prevalence as tracer conditions. These AMIs are monitored to establish the extent to which women with these conditions receive the appropriate major obstetric intervention (MOI). The underlying rationale is to assess, how a health system *actually *deals with presenting obstetric problems vis-à-vis how it *should *respond if it were functioning well. Our objective was to quantify the deficit in these life-saving interventions, or in other words the unmet obstetric need. Furthermore, we aimed at assessing urban-rural differentials, linking interventions to outcomes and evaluating the feasibility of the UON approach for health service management at district level.

## Methods

The UON methodology has been described in detail by the UON network [8]. In brief, absolute maternal indications (AMI) are defined as:

• Antepartum haemorrhage due to placenta praevia or abruptio placenta

• Malpresentation (transverse lie, brow presentation etc)

• Ruptured uterus

• Cephalo-pelvic disproportion/obstructed labour based on partograph with action line crossed by the dilation line (for the purpose of this study, this indication was intended to replace all other "indications" like poor progress, dystocia, prolonged labour etc.)

• According to Tanzanian national guidelines and expert opinion, more than two previous Caesarean Section were also included.

These conditions are selected not only because of their life-threatening nature, but also as they require a specific major obstetric intervention which can be verified through health service records. These major obstetric interventions (MOI) were adapted to the Tanzanian situation and included the following:

• Caesarean Section

• Hysterectomy following a caesarean section

• Laparatomy for obstetric interventions

• Destructive operation

• Blood transfusion during pregnancy or delivery.

Blood transfusion was included initially as a major obstetric intervention by the Tanzanian UON team; however, in the course of evaluating the data it became clear that it had not always been reliably recorded. It was therefore decided not to include this in the analysis of results. Therefore, the original UON concept remains as proposed by the UON network and is comparable to studies conducted in other countries. The UON network acknowledges that not all potentially life-saving interventions are included. To keep the concept operational the interventions selected were by necessity specific obstetric interventions such as are reliably recorded in routine registers, theatre ledgers, delivery books, and patient registers [8].

The UON network rests on the premise that as an absolute minimum 1–2% of pregnant women shall need a major obstetric intervention to save their lives [9]. This figure, based upon historical data from England and Wales [10], is supported by recent UON studies in other countries which have taken thresholds ranging from 1.0% to 1.6% [8]. Based on a Tanzanian pilot study in Mtwara urban district revealing a value of 2.4% [11], literature, and expert advice, the Tanzanian UON team decided to set this threshold for Tanzania at 2% of all deliveries.

### Study area and study population

The study area chosen was Tanga, a coastal region comprising six districts. Tanga Region has a population of 1,642,015. Three of the six districts took part in the study, namely Lushoto, Muheza and Tanga Municipality. Lushoto and Muheza are rural districts, in which the majority of the population lives in rural areas. Tanga Municipality is an urban district where 80% of the people are living in urban areas. In the study area the populations are 419,970 for Lushoto, 279,423 for Muheza and 243,580 for Tanga Municipality.

The study was conducted in 4 Hospitals; the Regional Hospital in Tanga; the District Designated Hospital in Muheza; the district hospital in Lushoto and in a church-run hospital in Bumbuli, Lushoto District. The methodology was explained to the key maternity staff from the four hospitals together with experts from Muhimbili National Hospital during a two-day workshop in Tanga resulting in the adaptation of the UON questionnaire to the local needs. Furthermore, it was decided to explore whether access to MOI varies according to rural or urban settings. Using the indicator from the 2002 census the distribution of the population was considered against the criterion that up to 10 km from the district main city is urban and >10 km is rural [12]. According to the above deliberation the population in rural areas is 88% in Lushoto, 89% in Muheza and 18% in Tanga Municipality.

### Data collection and analysis

In accordance with the study protocol a questionnaire was filled in for each of the above-mentioned major obstetric interventions carried out, based on the relevant documents (delivery record, antenatal card, theatre book, partograph) and information provided by health workers when needed. The questionnaire was signed by the trained staff member and later by the supervisor. It has to be noted that health workers of the participating maternities were intensively schooled on the importance of partograph use on a routine basis prior to the commencement of the study.

To estimate the UON per expected live birth a crude birth rate of 46 per 1,000 was used as is recommended practice of the Tanzanian Ministry of Health and Social Welfare. The percentage of MOIs per AMI per expected birth was calculated for the districts based on the demographic data from the National Census 2002 [12]. To estimate the deficit in MOIs as proxy for unmet obstetric need, a threshold of how many interventions are necessary in a given population has to be set. As stated, a threshold of 2% of all deliveries is used for this study.

The study has been conducted over a period of two years between 2000–2002. In Lushoto data was collected over a period of 27 months. All the questionnaires were sent to the regional headquarter in Tanga, where the data entry was undertaken. Altogether, completed questionnaires for 1,260 MOIs were received and analysed in EPI-Info 2000. Out of these, 905 (71%) were carried out for AMIs and were used to assess the unmet obstetric need.

As a measure of quality control the data derived from the UON questionnaires on caesarean section rates were cross-checked with data from the official health information and management system.

Concerning limitations, it has to be noted that when the questionnaire was adapted for the Tanzania context, "blood transfusion" was also considered to be a major obstetric intervention. However, the findings revealed that some women were apparently transfused during pregnancy but did not go on to deliver at that point meaning that certain parts of the questionnaire (maternal outcome/infant outcome) were not filled in. Thus, these questionnaires were not included in the analysis. It could also be the case that blood transfusions were not necessarily perceived as a MOI by health staff which might explain the lack of follow-up.

The comparison of the UON data and service data (Table 1) shows that reporting UON data from the districts is almost complete, while it appears that there was considerable under-reporting from Bombo regional hospital in Tanga. Therefore, our results from Tanga municipality may be an underestimate of MOIs and thus an overestimate of the unmet obstetric need.

**Table 1:** Comparison of Population-based Caesarean Sections Rates according to the Health Information Management System and Study Data

	**2000 HMIS % of reported C. Section deliveries**	**2001 HMIS % of reported C. Section deliveries**	**2002 HMIS % of reported C. Section deliveries**	**All Caesarean Sections (n = 1218) from study data as proportion of expected deliveries in one year**
**Lushoto**	1.0	0.8	1.3	1.1
**Muheza**	1.7	1.8	2.0	1.8
**Tanga Municipality**	2.7	2.7	3.7	1.5
**Total Region**	1.3	1.6	1.9	1.4

The study was initiated by the Muhimbili National Hospital and Muhimbili University College of Health Sciences, Dar es Salaam, and implemented in cooperation with the health authorities in Tanga region and the Tanzanian German Programme to Support Health. Ethical approval was obtained from the University College; further approval was obtained from the Regional Authorities in Tanga Region and the Management Teams of the participating hospitals. The information collected in the questionnaires related to the presenting indications and actions taken and did not include any personal data.

## Results

### Absolute Maternal Indications resulting in a Major Obstetric Intervention

Table 2 shows the most common AMI to be cephalo-pelvic-disproportion (CPD) which amounts to more than 50% of all the AMIs. Other common AMI include malpresentation or two or more previous caesarean sections.

**Table 2:** Absolute Maternal Indications resulting in a Major Obstetric Intervention

	**Region total**		**Lushoto**		**Muheza**		**Tanga Municipality**	
	
**Indications according AMI**	**N**	**%**	**N**	**%**	**N**	**%**	**N**	**%**
Cephalo-pelvic-disproportion	482	53.3	191	51.5	185	63.8	106	43.4
Malpresentation	193	21.3	101	27.2	43	14.8	49	20.1
2 or more previous C. Sections	135	14.9	35	9.4	30	10.3	70	28.7
Antepartum haemorrhage	61	6.7	28	7.5	22	7.6	11	4.5
Uterine rupture	34	3.8	16	4.3	10	3.4	8	3.3

**Total**	905	100	371	100	290	100	244	100

### Distribution of Major Obstetric Interventions in Tanga Region

In the 2 years study period 1,260 major obstetric intervention were reported; 467 from Lushoto, 457 from Muheza and 336 from Tanga as is shown in Table 3. Most of these (>95%) were Caesarean sections. The 905 MOIs based on AMIs represent approximately 71% of all MOIs. Thus, 29% of MOI are being conducted in response to "non-maternal indications" such as fetal distress (18.5% of all MOIs), breech presentation (5.1%), and cord prolapse (2.4%).

**Table 3:** Distribution of Major Obstetric Interventions in Tanga Region

	**Tanga Region (Total)**		**Lushoto**		**Muheza**		**Tanga Municipality**	
	
**Type of MOI**	**N**	**%**	**N**	**%**	**N**	**%**	**N**	**%**
Caesarean Section	1218	96.7	448	95.9	448	98	322	95.8
Hysterectomy	20	1.6	1	0.2	6	1.3	13	3.9
Laparotomy (for repair of ruptured uterus)	20	1.6	16	3.4	3	0.7	0	0.0
Destructive Operation	1	0.1	1	0.2	0	0.0	1	0.3
Others	1	0.1	1	0.2	0	0.0	0	0.0

**Total MOI**	1260	100	467	100	457	100	336	100

### Expected number of MOI for AMI vis-à-vis actual number performed

Table 4 shows that in all three districts the actual number of MOI for AMI is below the minimum benchmark of 2%. In addition there are differences across the districts: In Lushoto the expected number of MOI for AMI for the expected number of births is 0.8%, for Muheza 1.1% for Tanga Municipality 1.1% and for the whole Region 1%.

**Table 4:** Expected number of MOI for AMI vis-à-vis Actual Number Performed

	**Expected births (EB) per year**	**Need for MOIs for AMIs per year (2%)**	Actual number of MOI for AMI *	**Deficit in MOIs for AMIs**	**MOI for AMI for EB as %**
**Lushoto**	19,319	386	164	222	0.8
**Muheza**	12,853	257	145	112	1.1
**Tanga Municipality**	11,205	224	122	102	1.1
**Tanga Region**	43,286	867	431	436	1.0

*figure for a 12 month period calculated based upon study data for Lushoto this meant considering that data were collected for 27 months as opposed to 24 months for the other districts.

Given that the caesarean section is the MOI most often provided in the case of an AMI, it was considered useful to compare the study data with the data generated routinely on the number of caesarean sections performed overall.

### Overall caesarean section rate reported via Health Information Management System

In order to assess the completeness of our data a comparison was made to the caesarean section rate as recorded in the official Health Management Information System (HMIS) and shown in Table 5. HMIS figures show the percentage of caesarean deliveries for all six districts in Tanga Region. For 2000 the overall caesarean section rate in the region is 1.3%, in 2002 the figure is 1.9%.

**Table 5:** Comparison between Actual MOI for AMI in relation to Urban or Rural Residence and the Expected Minimum Level of 2%

	**All MOI for AMI per year (N)**	**Urban pop. (%)**	**MOI for AMI < 10km urban (N)**	MOI for AMI < 10 km as % of all deliveries* urban	**MOI for AMI >10 km rural (N)**	**MOI for AMI >10 km as % of all deliveries rural**
**Lushoto**	164	12%	56	2.4%	108	0.6%
**Muheza**	145	11%	69	4.9%	76	0.7%
**Tanga Municipality**	122	82%	105	1.1%	17	0.8%
**Tanga Region**	431	30%	230	1.8%	201	0.7%

*56 MOI for AMI were conducted for women living in urban areas of Lushoto according to study data, as a percentage of the expected EBs over one year for urban population of Lushoto (12% of 19,319) -see also table 4.

Overall the figures from the official HMIS and the study are comparable; however Tanga Municipality HMIS data indicates a higher caesarean section rate when compared to the study data. In addition, the HMIS data shows a slight overall increase in caesarean section rates between the years 2000 and 2002.

### Urban – rural differences in access to Major Obstetric Interventions

According to the reflection in the methodology, 11% of the pregnant women in Muheza in need of a caesarean section would live in the urban area and 89% in the countryside. However, the analysis of the data paints a picture that is very different. There is an important gap regarding the availability of services when urban and rural areas are compared. In the group of women living in areas classified as urban, and thus residing within 10 km of the hospital, the 230 MOIs for AMIs represent 1.8% of all deliveries and lies close to the 2% benchmark. The 201 MOIs in rural women represent only 0.7% of all deliveries.

### Perinatal and maternal outcome

Out of the 1,260 reported deliveries resulting in a MOI, regardless of the indication, 1,145 babies were born and discharged alive (90.9%), 86 of the babies were still born (6.8%). A further 22 were born alive but died within 24 hours of delivery (1.7%), whilst 6 survived the first 24 hours after delivery but died within the first week of their lives (0.5%). Thus the overall perinatal mortality was 9.1%. There were differences between the various districts, in Lushoto the percentage of still borns in relation to a MOI was 8.1%, in Muheza 6.3%, whereas in the Municipality it was 5.7%.

Regarding the maternal outcome of the 1,260 reported MOI 1,156 (91.7%) survived the intervention with no adverse effects, 70 women developed a complication such as haemorrhage, infection, anaesthetic complication, severe anaemia, hypertension, cardiomyopathy or herbal intoxication (6.8%), whereas 19 women died (1.5%). One woman was referred, but it was not possible to follow up the outcome. Similarly, there were differences between the hospitals with 94.4% of women in Lushoto having no further complications, compared to 89.7% in Muheza.

## Discussion

Referring to our specific objectives, the study revealed that in cases where the woman has undergone a major obstetric intervention the perinatal outcome is particularly poor with a mortality of 9.1%. The results further show a high maternal mortality of 1.5% and considerable morbidity, although there are variations between the hospitals. This situation may be due in part to delays in performing the necessary obstetric interventions but can also be seen to point to deficiencies in the technical quality of care provided. Thus, improving access to obstetric care needs to go hand in hand with monitoring and improving the quality of care of obstetric units.

An efficiently functioning district health system should be capable of providing medical care to all patients in need, regardless of the distance between their homes and the hospital. However, in all three districts a discrepancy is observed in the actual and expected distribution of MOI for AMI performed between women coming from urban and rural areas. When compared with the 2% threshold selected for the UON-indicator the study data reveal significant unmet-obstetric need in all three districts.

The findings show an overall MOI for AMI rate of 1% and compare to results from UON concept studies in other countries [10,13]. Despite the fact that in Tanga Region a core provision of essential obstetric care is in place, concerns about the accessibility of such services – including of referrals and blood transfusions – for rural women are raised and need to be addressed. Data from selected population-based studies in sub-Saharan Africa also shows the substantial differences in maternal mortality between urban and rural areas. Differences in physical access to obstetric care certainly explain the variation to an extent. The low proportion of MOI performed even in urban areas suggests, however, that the quality of service provision is also an important factor [7].

Given that so many women in Tanzania attend ANC, there is a clear need for its refocusing to increase its impact on maternal health and to link it to appropriate delivery care. The risk approach has also been shown to have only limited effectiveness. Detection and management of sexually transmitted diseases offer improvements in health without necessarily any equivalent reduction in the risk of maternal death [14]. The overall poor health of Tanzanian women should be remembered – nutritional status is often low whilst the total fertility rate remains high at 5.8 births per women for the period 1993–96 [4]. In many developing countries HIV infection in pregnancy has become the most common complication. The relative impact of HIV infection vis-à-vis inadequate antenatal care and adverse pregnancy outcomes is however difficult to separate out [15]. A study in Rakai district of Uganda found maternal mortality to be five times higher in HIV-infected than in HIV-uninfected women [6].

ANC contacts represents not only the ideal time to explain the benefits of facility-based deliveries and of post-natal care (where advice on further pregnancies can be offered), but also to discuss the issue of HIV-testing, the prevention of vertical transmission (from the mother to the child during pregnancy, delivery and lactation) and the provision of care and treatment should the parents be found to be HIV positive.

Progress towards the goal of preventing maternal death depends upon strong health systems [6]. There is a need for Essential Obstetric Care to be accessible – this means that it needs to be physically available, within reach both in terms of distance and financially, culturally acceptable, manned by skilled staff with the services they provide of recognized quality. The need for targeting of interventions towards the most vulnerable groups (mostly rural populations and the poor) as part of an overall targeting of improvements in measuring the burden of mortality in these groups is current consensus [6]. This touches upon the issue of accountability as Governments pledge to overcome inequities in health care provision.

Whilst the results indicate a need for better access to caesarean sections, efforts need to avoid the risk of raising the number of *unnecessary *caesarean sections and of iatrogenic morbidity and mortality [16]. Support should be part of a global effort towardappropriate use of caesarean sections – limiting the intervention's frequency where it is overused and increasing it where use istoo low.

Referring to specific observation during the course of the study, we have noted that giving importance to the indications, as required by the UON approach, has greatly improved the completeness and quality of documentation of obstetric interventions. This extended to the use of the partograph to substantiate the diagnosis of CPD, which accounted for about half of all major obstetric interventions. The data were useful for the yearly district planning and UON data could be analysed in detail at district level. The comparison of the UON data with aggregated district data on caesarean sections showed that the UON data collection was complete in two districts but incomplete in Tanga Municipality, resulting in a likely underestimate of MOIs there. The problems of including blood transfusions in the list of MOIs clearly showed that data collection in busy hospitals needs to rely on well established routine records. The lack of a standardised way to document and register pregnancy-related blood transfusions was the main reason why the inclusion of this intervention could not be pursued at this stage. However, a facility-based documentation of blood transfusion, similar to the documentation of surgical interventions, could be considered.

## Conclusion

Tanga Region, with an overall Major Obstetric Intervention for AMI rate of 1% and a caesarean section rate of 1.4% among all expected births, has important unmet obstetric need when the study results are compared with the 2% threshold.

There is a considerable discrepancy when the expected and actual MOI for AMI are compared to whether the woman comes from an area classified as rural or urban. The low level of MOI for AMI (0.7%) amongst the rural population points to major unmet obstetric need there. The high proportions of negative outcomes in mothers undergoing a MOI (complications 6.8%; mortality 1.5%) and in new-borns (perinatal mortality 9.1%) raise concern about the quality of care being provided.

The UON concept has shown itself to be a tool that can be handled at district level with little additional support beyond small incentives for those who took part in filling out the questionnaires. The data can be used for planning and to monitor trends in the responsiveness of the health system at intervals. Of particular importance is that the data is generated by stakeholders in the systems, who share optimism that the findings can revitalize the discussions on access to obstetric care at the national level in Tanzania.

## Competing interests

The author(s) declare that they have no competing interests.

## Authors' contributions

SM, FM, and AJ developed the concept and the study design, including the adaptation of the UON methodology to the Tanzanian context. HP and FM organised and supervised the data collection. HP did the data analysis and interpretation, supported by RK, AJ and CH. HP drafted the paper jointly with RK, which was then critically reviewed and revised by SM, AJ and CH. All authors read and approved the final manuscript.

## Pre-publication history

The pre-publication history for this paper can be accessed here:


